# Experimental Study on Effect of Simulated Microgravity on Structural Chromosome Instability of Human Peripheral Blood Lymphocytes

**DOI:** 10.1371/journal.pone.0100595

**Published:** 2014-06-25

**Authors:** Lijun Wei, Chuanpeng Liu, Li Kang, Yufeng Liu, Shuliang Shi, Qiong Wu, Yu Li

**Affiliations:** 1 School of Life Science and Technology, Harbin Institute of Technology, Harbin, China; 2 State Key Laboratory of Space Medicine Fundamentals and Application, Chinese Astronaut Research and Training Center, Beijing, China; Texas Tech University, United States of America

## Abstract

Experimental study was made by keeping human peripheral blood lymphocytes under simulated microgravity in a Rotary Cell Culture System bioreactor to investigate the changes that occur in the number of chromosomes, the expression rate of chromosome fragile site, and the expressions of DNA replication- and repair-related genes. Experimental results indicate simulated microgravity has no effect on the numerical chromosome instability of human peripheral blood lymphocytes, but it enhances the structural chromosome instability of human peripheral blood lymphocytes through the inhibition of DNA replication and the reduction of DNA repair. So, the mechanism of chromosome fragile site induced by simulated microgravity can be explained using the changes that occur in the chromosome structure of human peripheral blood lymphocytes, the DNA replication and repair under the effect of simulated microgravity.

## Introduction

Chromosome instability (CIN) is a great concern for manned-space flight because of the space environment-induced damages to human chromosomes observed in the lymphocytes of Astronauts [1]. Structural and numerical chromosome instabilities are the two aspects of CIN commonly observed in solid tumors [2], [3]. The numerical chromosome instability is known as aneuploid, and the structural chromosome instability means deletion, inversion, translocation and rearrangement of chromosomes [4], [5]. Chromosome fragile sites are specific loci that preferentially exhibit gaps and breaks in metaphase chromosomes after DNA synthesis is partially inhibited [6]. Thus, breaks may cause deletion of chromosome, fragmentation without centromere and enhancement of chromosome aberration [7]. So, the expression of chromosome fragile site is closely related to chromosome aberration.

About 120 fragile sites have been described so far. They are normally stable in cultured cells, but they form visible gaps and breaks in metaphase chromosomes under certain culture conditions or by treatment with specific chemical agents. These fragile sites can be generally categorized into two classes of common and rare fragile sites according to their population frequencies and patterns of inheritance [6]. There are about 90 common fragile sites listed in the Genome Database (GDB) at present [6].

Common fragile sites are normally stable in somatic cells. However, they display gaps, breaks, rearrangements and other features of unstable DNA, which is referred to as the expression of fragile site, when the cells are cultured under folate or thymidylate stress or with a low dose of aphidicolin to partially inhibit DNA synthesis [7], [8]. The expression of fragile site is the result of a cell which escapes from the cell cycle checkpoint and then forms a DNA single- or double strand break. The cell cycle can be arrested and the unreplicated chromosomal regions can be repaired if the cell cycle checkpoint ATR is activated and DNA replication is stopped [9], [10].

Microgravity in space is a kind of culture stress which can induce the inhibition of cell proliferation, the occurrence of cytoskeleton disorder, the structural change in a bipolar spindle, the arrestment of a cell cycle and/or the alteration of a gene expression. However, little work is done on the chromosome instability of human peripheral blood lymphocytes (PBL) under space microgravity or simulated microgravity.

Therefore, our study on the effect of simulated microgravity is continued by keeping human PBL cells under simulated microgravity in a Rotary Cell Culture System (RCCS) bioreactor to investigate the effect of simulated microgravity on the structural chromosome instability of PBL cells. This article is a summary report on our recent work to complete our study on the effect of simulated microgravity, and it reports new findings that extend the results we reported earlier [11].

## Material and Methods

### Ethics Statement

The 20 samples of human PBL cells used for the present study were provided by The Second Affiliated Hospital of Harbin Medical University. The use of these PBL cells is approved by the Ethical Committee of the Second Affiliated Hospital of Harbin Medical University in accordance with the ethical standards laid down in the 1964 Declaration of Helsinki for researches involving human subjects. The written consents of all participating subjects were obtained prior to the commencement of the study.

### Culture of PBL cells

The human PBL cells used for the present study are taken from healthy male donors within an age range of 25–35 years, and are stored in anticoagulation tube at 4°C no more than 24 hours before analysis. The cells are cultured in RPMI-1640 (Gibbco, BRL) with 20% (v/v) fetal calf serum (FBS, Gibbco BRL), 25 µg/ml PHA (Sigma) and antibiotics (streptomycin: 100 µg/ml; penicillin G: 100 U/ml). The cells are incubated at 37°C in a humidified atmosphere containing 5% CO_2_.

### Cell culture in rotating bioreactor

Simulated microgravity culture conditions are created using a Rotary Cell Culture System (RCCS) bioreactor, which is a useful tool to detect the effect of simulated microgravity on a variety of mammalian cells in vitro on ground [12]–[15]. It is reported that the cells growing in a RCCS bioreactor underwent the similar morphological and functional changes compared to the cells in space flight [12]–[15].

The human PBL cells which are stored in anticoagulation tube are directly seeded in the bioreactor vessel with a diameter of 0.1 m with a density of 5×10^5^ per ml. According to the equipment manual, the samples are rotated around the horizontal axis at 10 rpm, and the residual accelerations are less than 10^−2^ *g*. The cells cultured under the earth's gravity are considered as the untreated control.

### Number of chromosomes and Mitotic Index analysis

The PBL cells are harvested by centrifugation at 114 g for 5 minutes after they are kept under simulated microgravity for 72 hours. Before harvesting, they are treated with colchicines(0.02 µg/ml)for 1 hour. The cells are fixed with fresh methanol and glacial acetic acid fixative (3 vol: 1 vol) (Sinopharm chemical reagent Co. Ltd, China) for 30 minutes. The fixative is changed twice during the cell fixation. A drop of suspension is put onto the slide and stained with 10% (v/v) Giemsa (Sigma, USA). The numbers of chromosomes are counted with coded (blinded) slides scored under a BX41 Olympus microscope at 400× magnification. The cells undergo the same process of chromosome observation for MI analysis. From the total number of cells and the number of mitotic cells counted, MI is calculated using the following formula: MI (%)  =  (number of mitotic cells/total number of cells) ×100%.

### Expression rate of chromosome fragile site

Common fragile site is usually induced by culturing PBL cells in folate-free or aphidicolin added medium for medical diagnosis. These two methods are used to obtain clear gaps for the present study. The blood collected from volunteers is cultured in folate-free M199 medium (5% FBS, pH 7.5), 25 µg/ml PHA (Sigma) and antibiotics (streptomycin: 100 µg/ml; penicillin G: 100 U/ml), and 0.4 µM aphidicolin is added to the medium 26 hours before cell harvesting. The cells are harvested through the same process of chromosome observation. Coded (blinded) slides are scored under a BX41 Olympus microscope at 1000× magnification. 100 mitotic images are collected for each sample. The numbers of cells observed and the numbers of chromosomes with fragile sites are counted according to nonstaining chromosome, chromatid gaps or chromosome breakpoint. The expression rate of chromosome fragile site is calculated using the following formula:Chromosome fragile site expression rate (%)  =  (number of chromosome fragile sites occurred/number of chromosomes observed)×100%.

### Determination of chromosome fragile sites using G-banding technique

G-banding technique is used to further confirm chromosome fragile sites. The metaphase chromosomes are treated with trypsin (to partially digest protein) and stained with Giemsa. Trypsin solution is prepared by dissolving 25 mg trypsin in 50 ml of 0.85% NaCl, and 2 drops of 0.4% Phenol-sulfonphthalein are added, and pH is adjusted to 6.4–6.6 with 5%NaHCO_3_. Oven-dried slides are dipped in the trypsin solution for 3–7 seconds. The slides are rinsed in water and then stained in a coplin staining jar with Giemsa for 8–10 minutes. The slides are rinsed in water and then checked using a microscope at 100× oil objective.

A “normal” human carries 23 pairs of chromosomes, and there are 7 chromosome groups according to their G-banding types, and each chromosome has its special pattern [16].100 mitotic cells are analyzed, and chromosome fragile sites are determined against the international standard.

### Analysis of ATR Gene Expression by qRT-PCR

The PBL cells at logarithmic growth phase are treated with a rotating wall vessel bioreactor, and after the treatment, the cells are harvested by centrifugation (114 g for 5 min). The total RNA of the treated and the control cells is extracted with TRIzol Reagent (Invitrogen, USA) in accordance with the manufacturer's instructions. And 1 µg of the total RNA is reverse-transcribed into cDNA using High Capcity cDNA Reverse Transcription Kit (Applied Biology, USA) with random hexamer primersand and multiscribe reverse transcriptase. The cDNA samples are stored at −20°C for further test.

Quantitative real-time PCR is performed in accordance with the MIQE guidelines [17] using the Applied Biosystems 7500 Real-Time PCR system together with the SYBR Premix Ex Taq (Perfect Real Time) (Takara). The GAPDH is used as an internal control in quantitative real time RT-PCR with SYBR green dye (SYBR Premix Ex Taq Perfect Real Time Kit). The primer sequences are: ATR forward 5′-TTACAATGAGGCTGATGCG-3′ and reverse 5′- TGCCCTTTCACTGGTTTACT-3′; GAPDH forward 5′-AACAGCCTCAAGATCATCAGC-3′ and reverse 5′-GGATGATGTTCTGGAGAGCC-3′. PCR conditions are: 95°C for 10 second, 95°C for 5 second, 60°C for 34 second for 35 cycles. Reaction specificity was controlled through post-amplification amplification melting curve analyses. The copy number is obtained using 2-ΔΔCT method [18]. The relative expression levels of ATR are normalized to the amount of GAPDH. The expression of ATR gene in 96-well plates is quantified using an iCycler iQTM Multicolor Real-Time Detection System (Bio-Rad, USA).

### Microarray analysis

The PBL cells are harvested after they are kept under simulated microgravity for 72 hours, and 2×10^6^ cells of each sample are mixed to establish a PBL cell pool, and these cells are stored in TRIzol Reagent (Invitrogen, USA). The samples are sent to CapitalBio Corporation, and the gene expression profile is analyzed using human genome oligonucleotide microarray V2.0. The raw data of mRNA expression profile is analyzed using LuxScan 3.0 software and CBC Analyzer to obtain the difference of gene expression and *P* value. The threshold between differentially expressed genes are set with Fold Change ≥2. The results of array are verified using qRT-PCR technique. The dataset are submitted to Gene Expression Omnibus, and the accession number is GSE56647.

### Statistical analysis

Chi-square test (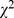-test) is used to test the distribution differences between the chromosome number, MI and chromosome fragile site expression number. A *P*-value of less than 0.05 is considered as a statistical significance indicator. The data shown are obtained through three independent experiments.

## Results and Discussion

### The number of chromosomes does not change under different culture conditions, and so, simulated microgravity has no effect on the numerical chromosome instability of human PBL cells

**Figure 1:**
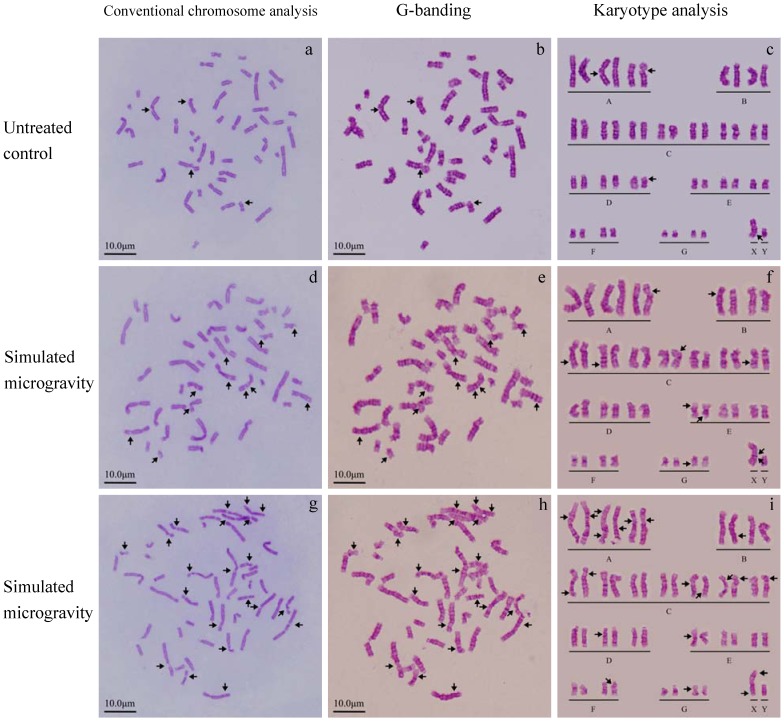
Karyogram with chromosome arranged into a standardized format. G-banding and karyotype analysis were used to further confirm the changes of chromosomes in number and structure either in untreated control group (line 1) or simulated microgravity group (line 2 and 3). Chromosome breakages are marked with arrows. The karyotypes are the results of further analysis based on G-banding indicated there was neither chromosome gain or loss, nor deletion, inversion and/or translocation of chromosomes.

Two different culture media, RPMI-1640 with 20% FBS, and folate-free M199 medium with 5% FBS and 0.4 µM aphidicolin were used under 1 *g* or simulated microgravity for the present study. In order to investigate the environmental influence on the chromosome segregation of human PBL cells, the number of chromosome was determined first under different culture conditions. As shown in Fig.S1a-d and Table 1, the total number of chromosomes did not show any significant change. G-banding technique and karyotype assay were used to further confirm whether there was any gain or loss of a whole chromosome. As shown in Fig.1, there is no chromosome gain or loss of a whole chromosome. It can be seen from the research results that none of the culture conditions used for this study shows any effect on the chromosome segregation of human PBL cells, i.e. simulated microgravity has no effect on the numerical chromosome instability of human PBL cells. It can therefore be concluded that the numerical chromosome instability of human PBL cells is not enhanced by simulated microgravity.

**Table 1:** Number of chromosomes kept under simulated microgravity for 72

Medium	Sample No.	Treatment	Total number of cells detected	Number of cells with 46 chromosomes	*P*
RPMI-1640 with 20% FBS	1	CK	100	96	0.516
		SMG	100	94	
	2	CK	100	95	0.552
		SMG	100	93	
	3	CK	100	91	0.421
		SMG	100	94	
	4	CK	100	93	0.788
		SMG	100	92	
	5	CK	100	94	0.579
		SMG	100	92	
Folate-free M199 medium with 5% FBS	6	CK	100	94	0.579
		SMG	100	92	
	7	CK	100	95	0.552
		SMG	100	93	
	8	CK	100	96	0.234
		SMG	100	92	
	9	CK	100	95	0.268
		SMG	100	91	
	10	CK	100	92	0.389
		SMG	100	95	

CK- Untreated control.

SMG- Simulated microgravity.

### After the cells were kept in serum added 1640 medium under simulated microgravity for 72 hours, the chromosome fragile site does not occur but the expression of ATR is enhanced and the proliferation of cell is inhibited

The structural chromosome instability of PBL cells was tested after the cells were kept in serum added 1640 medium under simulated microgravity for 72 hours. As shown in Fig.S1a and b, no chromosome breakpoint (chromosome fragile site) was observed, which meant that microgravity has no effect on the increase in the expression of chromosome fragile site of human PBL cells. However, no change was observed possibly because of the completed self-repair of a damaged cell. So, the expression of ATR, the arrestment of cell cycle and the change in mitotic index were further analyzed.

*ATR* is an important factor having effect on the DNA damage pathway, and it can be used to detect the occurrence of a gap during the replication of DNA [9], [10] and the up regulation of the expression to block the cell cycle and to complete the cell repair. Glover et al. (2005) confirmed that the expression of *ATR* is enhanced as the replication of DNA is inhibited, i.e., the breakpoints increase as the expression of *ATR* increases [9].

**Figure 2:**
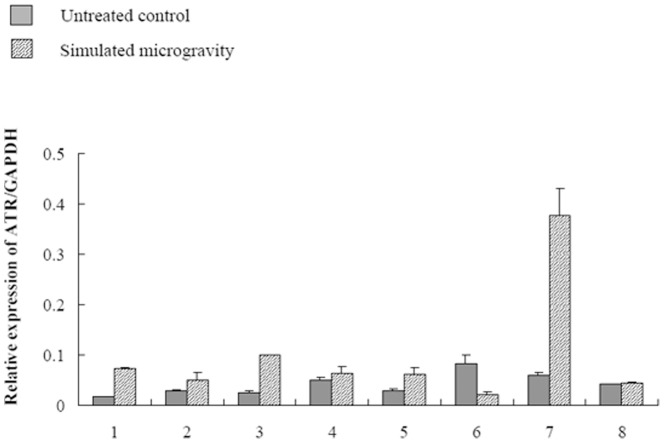
Changes in ATR expression in 8 samples after the cells were kept under simulated microgravity for 72 hours. Quantitative real-time PCR was used to analyze the expression of ATR, and the expression of ATR becomes more than 1.5 times larger in samples 1,2,3,5 and 7, and a slightly larger in samples 4 and 8, while smaller in sample 6 after the cells were kept under simulated microgravity for 72 hours. The expression of ATR increases in most of human beings. The relative expression rate in mean± S.E. (n = 3).

**Figure 3:**
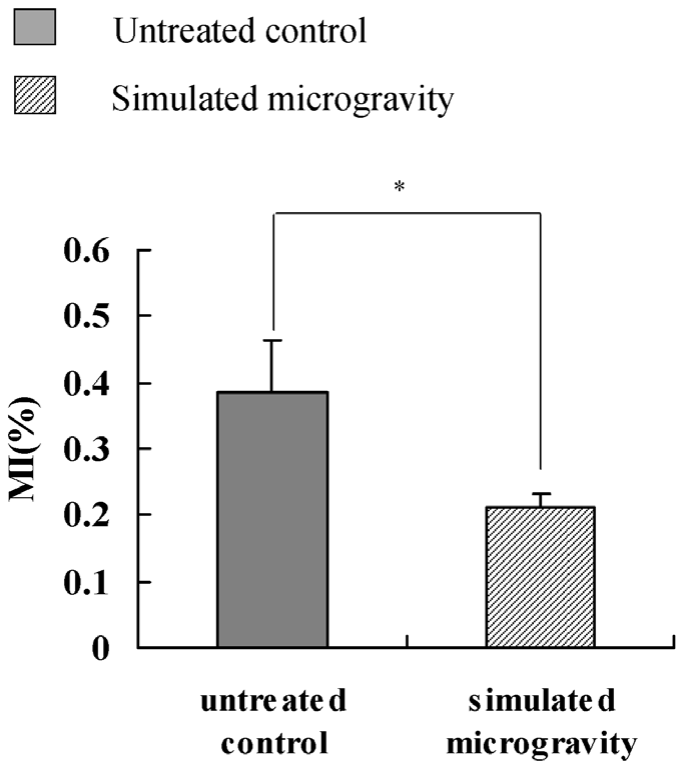
Significant decreases in Mitotic index of PBL cells were kept under simulated microgravity for 72 hours. The data represents mean±S.E. (n = 3). *0.01<*P*<0.05 and **P<0.01 (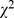-test as compared to control group).

Eight PBL samples were taken from healthy male donors and cultured in serum added RPMI-1640. The cells were harvested after they were cultured under simulated microgravity for 72 hours, and quantitative real-time PCR was carried out as mentioned above. As shown in Fig.2, compared to untreated control, after being kept under simulated microgravity for 72 hours, the relative expressions of ATR in sample No.1–8 was 4.03, 1.76, 4.20, 1.25, 2.20, 0.26, 6.40, 1.07 times that of untreated control, respectively. This means that the expression of *ATR* increases in most of human beings, as the inhibition of DNA replication further increases. It was found in our early study that the cell cycle arrested at S phase in human immortalized lymphocytes after the cells were kept under simulated microgravity for 72 hours [19], which proved from another angle that the synthesis of DNA had been inhibited. Mitotic index was analyzed to further confirm the inhibition of PBL cells proliferation. As shown in Fig.3, after the cells were kept under simulated microgravity for 72 hours, the number of mitotic cells decreases significantly, which meant the cell proliferation was inhibited.

It can therefore be concluded that the inhibition of replication under simulated microgravity may cause the increase in the expression of cell cycle checkpoints to arrest the cell cycle, and to complete the repair of damage to DNA, and so, the chromosome fragile site can not be detected. After the cells were kept under simulated microgravity for 72 hours, the structure of DNA could be damaged, but the damage could be repaired by the cell itself.

### Enhancement of expression rate of chromosome fragile site in conditioned medium under simulated microgravity

**Figure 4:**
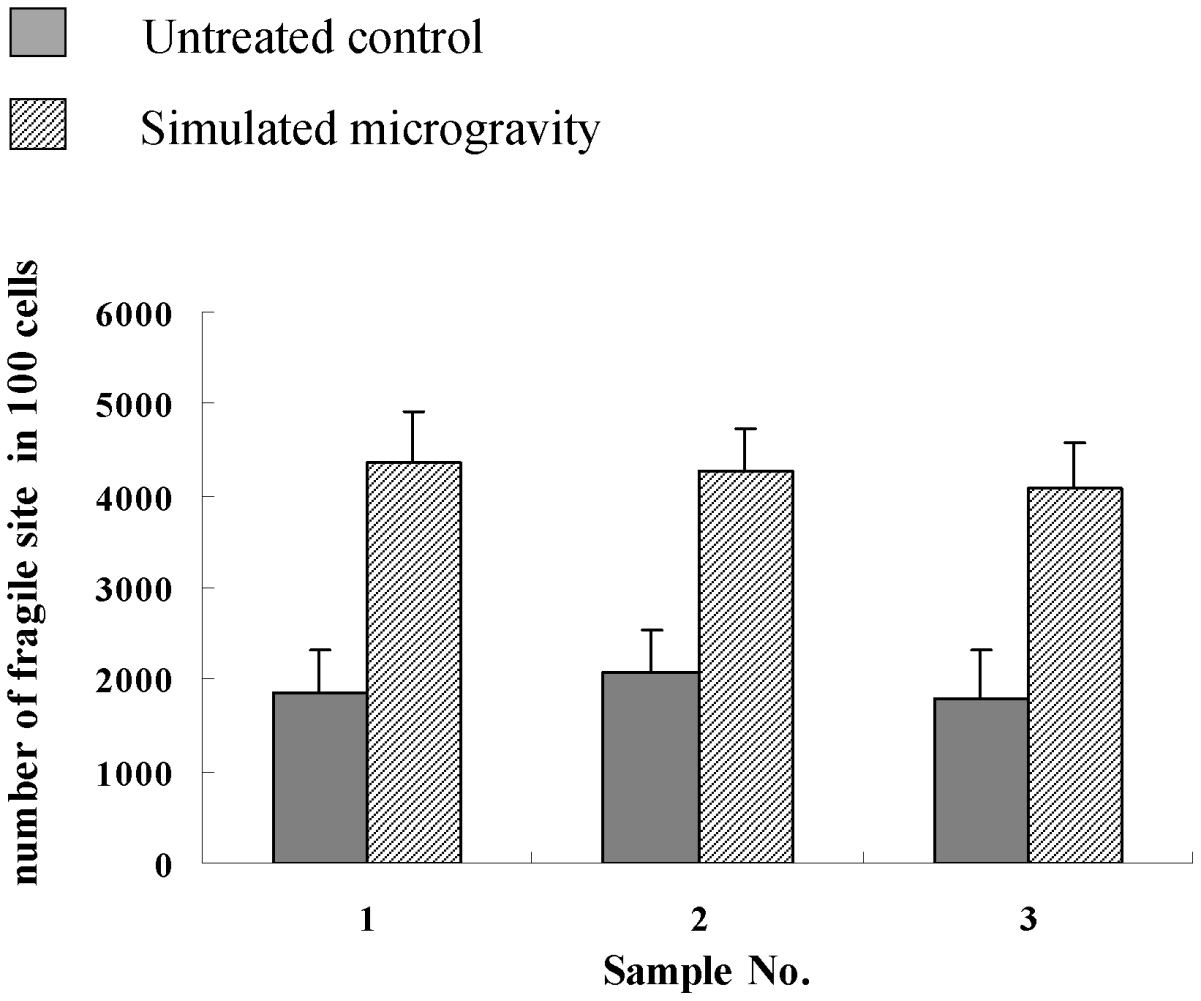
Number of chromosome fragile sites in 100 cells of 3 independent samples. The PBL cells were cultured in folate-free M199 and 0.4 µM aphidicolin added medium. 100 mitotic images of were collected from each sample, and both the number of cells observed and the number of chromosomes with fragile sites were counted. The chromosome fragile sites are determined according to nonstaining chromosome, chromatid gaps or chromosome breakpoints. The number of chromosome fragile sites is much higher for the cells after the cells being kept under simulated microgravity for 72 hours than that of the untreated control group for the three independent samples. *0.01<*P*<0.05 and **P<0.01 (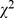-test as compared to control group).

A special culture medium or medicine was used to make the observation of chromosome breakages easy for the present study. The medium usually used for medical study is a folate-free M199 medium with low serum (5% of FBS) or RPMI-1640 with 20% FBS and aphidicolin, a medium with DNA replication inhibitor. The fragile site can be considered to be successfully induced when FRA3B is observed [20], [21]. And the presence of an expression of fragile site means the repair of DNA is below the actual damage degree of DNA. So, the inhibition of DNA replication can be identified through the observation of the change in chromosome morphological structure. This is the theoretical basis for our study on the effect of simulated microgravity on the replication of DNA and the structural chromosome instability of human PBL cells. As shown in Fig.1c-d, Fig.4 and Table S1, the expression rate of chromosome fragile site had no significant difference among the three individuals, but it had a significant increase after the cells were kept under simulated microgravity for 72 hours. This meant simulated microgravity enhance the inhibition of DNA replication and induces the change in chromosome structure. It can be seen from the morphologic changes that more gaps occur in larger chromosomes.

**Figure 5:**
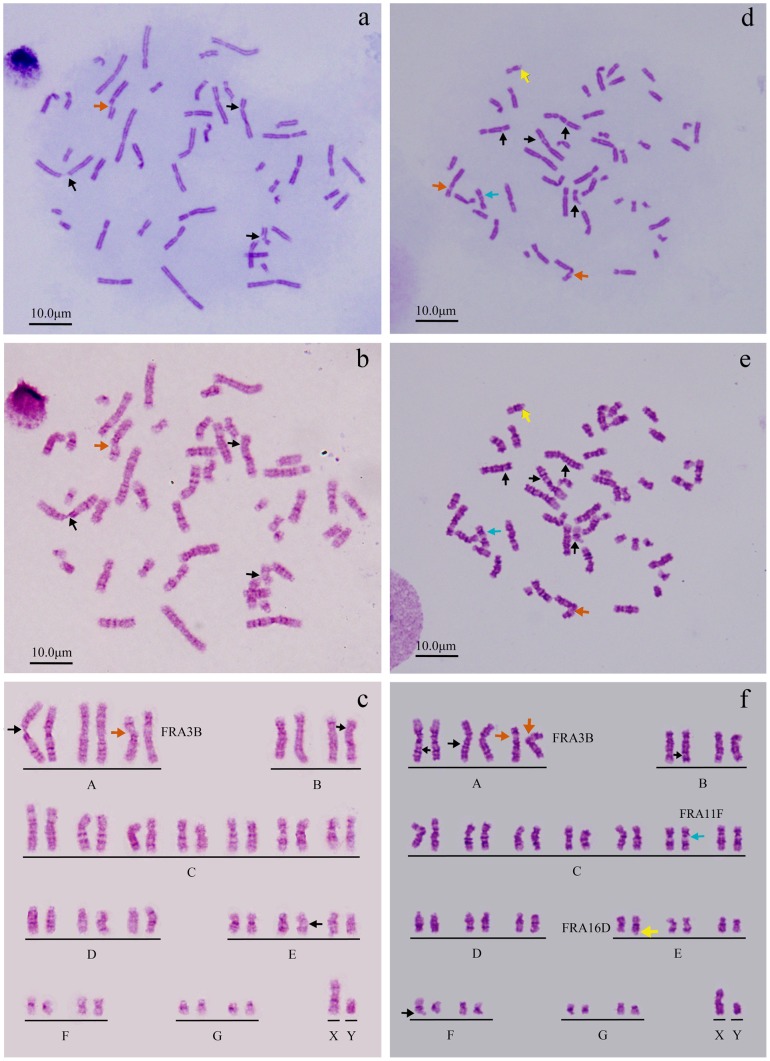
Loci of chromosome fragile sites were determined using G-banging technique through karyotype analysis of PBL cells of sample No.2. a-c. untreated control, d-f. simulated microgravity for 72 hours. Chromosome morphology was analyzed using conventional chromosome analysis technique (a and d), and the break point (arrow) can be clearly observed. Both G-banging technique and karyotype analysis were used to further confirm the loci of chromosome fragile site (b, c, d and e). The chromosome fragile sites on chromosomes 1, 2, 3, 11, 16 and 19 are highlighted by arrows by black arrows, except FRA3B by orange arrows, FRA11F by blue arrows, and FRA16D by yellow arrows.

**Figure 6:**
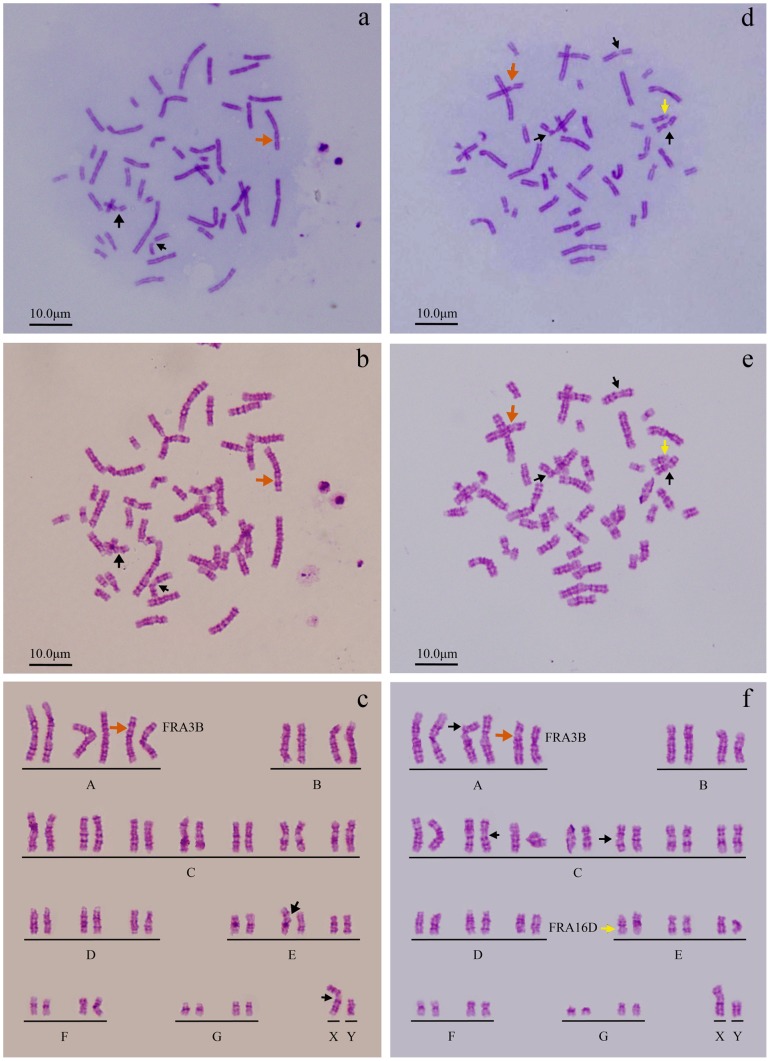
Loci of chromosome fragile sites were determined using G-banging technique through karyotype analysis of PBL cells of sample No.4. a-c. untreated control, d-f. simulated microgravity for 72 hours. Chromosome morphology was analyzed using conventional chromosome analysis technique (a and d), and the break point (arrow) can be clearly observed. Both G-banging technique and karyotype analysis were used to further confirm the loci of chromosome fragile sites (b, c, d and e). The chromosome fragile sites on chromosome 2, 3 7, 10, 16, 17 and X are highlighted by black arrows, except FRA3B by orange arrows, and FRA16D by yellow arrows.

**Figure 7:**
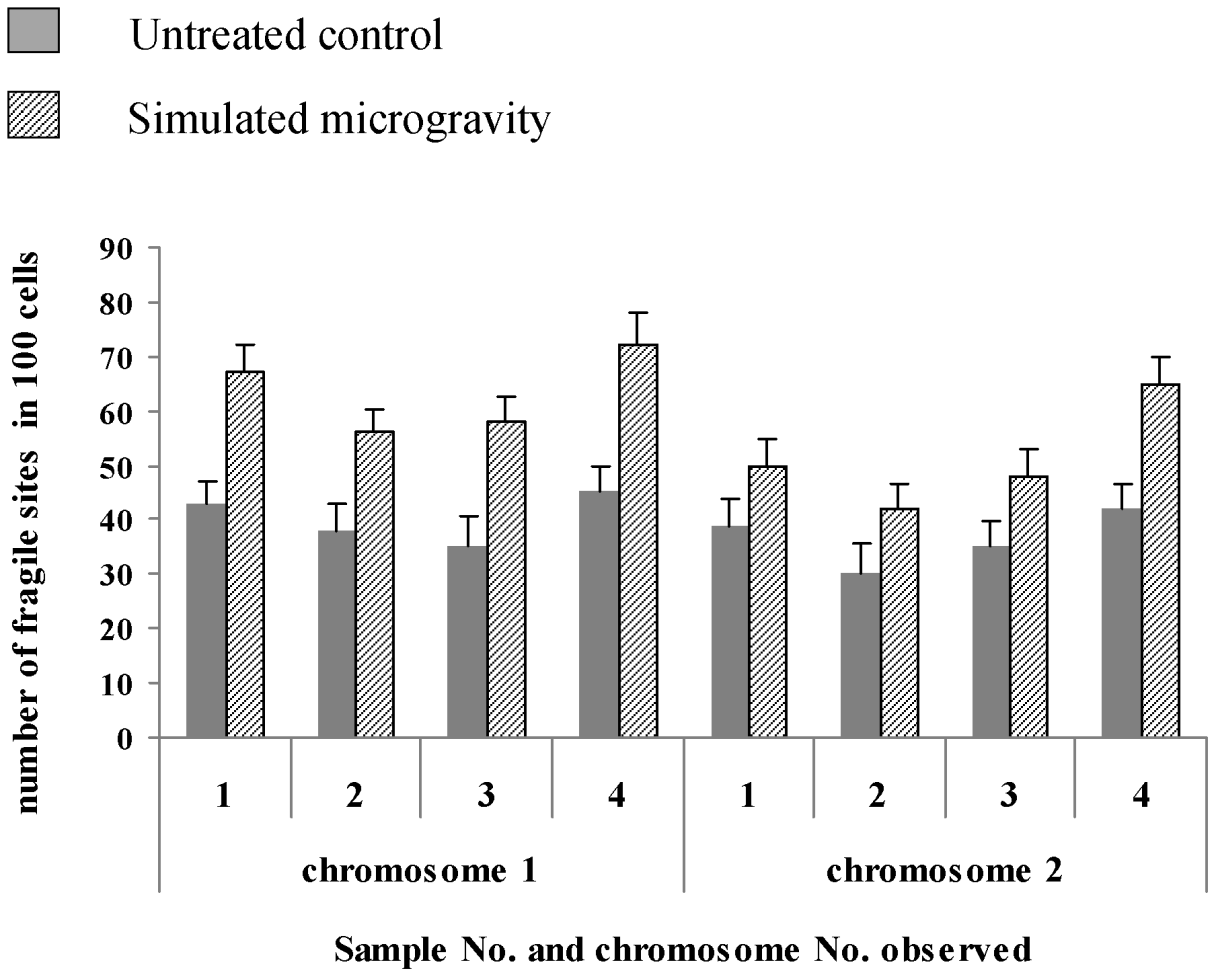
Number of chromosome fragile sites on chromosomes 1 and 2 of 4 independent samples. 100 mitotic images were collected from each sample, and both the number of cells observed and the number of chromosomes with fragile sites were counted. The number of chromosome fragile sites on chromosomes 1 and 2 is much larger for the cells after the cells were kept under simulated microgravity 72*0.01<*P*<0.05 and **P<0.01 (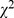-test as compared to control group).

**Figure 8:**
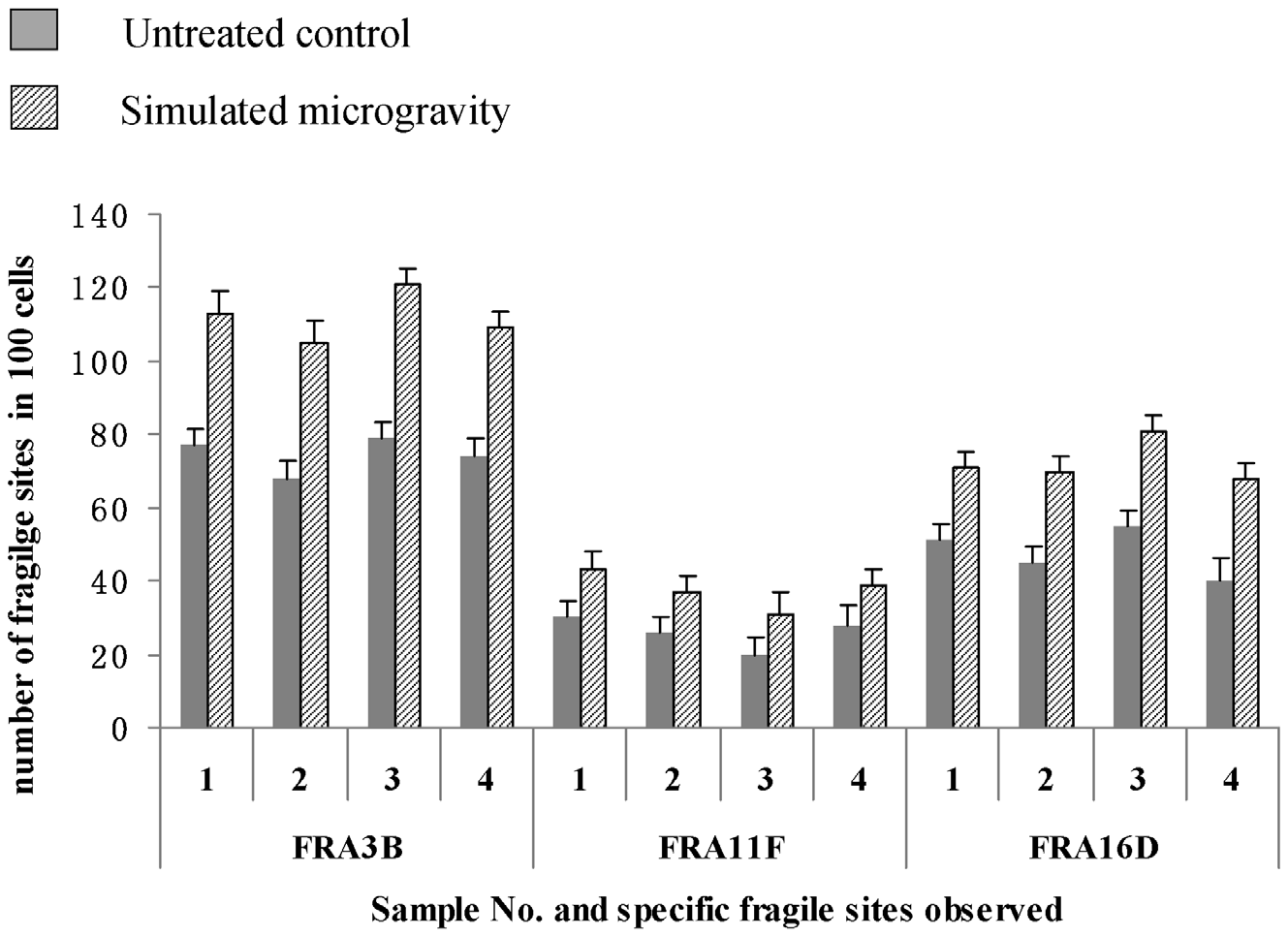
Expression rates of FRA3B, FRA11F and FRA16D of 4 independent samples. 100 mitotic images were collected from each sample, and both the number of cells observed and the number of chromosomes with fragile sites were counted. The expression rates of FRA3B, FRA11F and FRA16D increase after the cells were kept under simulated microgravity for 72*0.01<*P*<0.05 and **P<0.01 (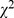-test as compared to control group).

G-banging technique was used to further confirm the break point of replication (Fig. 5 and Fig.6). It can be seen from Table S1 and S2 and Fig. 7 and 8 that the number of breakpoints in chromosomes 1 and 2, and the expression rates of chromosome fragile sites FRA3B, FRA11F and FRA16D increase under simulated microgravity. Among the three special loci, the expression rate of FRA3B is the highest,while that of FRA11F is the lowest, which is only half of that of FRA3B. It can be seen from these results that the occurrence of breakpoints in the chromosomes is not random. The results of G-banding and karyotype analyses also indicated that there was no deletion, inversion and/or translocation of chromosomes (Fig.1, 5 and 6).

### Reduction of expression of DNA repair gene under simulated microgravity

mRNA expression was analyzed by Bioassay Laboratory of CapitalBio Corporation with Human genome V2.0 oligonucleotide microarray, and the accuracy of array was verified using qRT-PCR technique (Table S3). Totally, 37593 genes and 170 pathways were detected using this array. Compared with untreated control(ratio value≥2.0 or≤0.5), the expressions of 208 genes showed significant changes, which include 119 genes up regulated and 89 genes down regulated. These genes are related to 49 pathways under simulated microgravity. Most noticeable pathways related to cell proliferation and DNA damage and repair (*P*<0.001) are tabulated in Table 2. DNA replication- and repair- related genes were further analyzed to explain the relationship between the expression of gene and fragile site. It can be seen from Table 3 that three genes *P11388-2*, *TYMS* and *PCNA* with the functions of DNA replication and repair are down regulated. Six genes *RFC2*, *GMNN*, *MCM7*, *FEN1*, *POLE3* and *GINS2* with the function of DNA replication are down regulated, and DNA damage response gene *BBC3* is up regulated.

**Table 2:** List of pathways with *P*<0.001 under simulated microgravity.

Pathway	*P*-Value
Apoptosis	2.35E-12
p53 signaling pathway	1.57E-11
Cell cycle	1.14E-06
DNA polymerase	1.48E-04
Oxidative phosphorylation	1.76E-04
Purine metabolism	3.43E -04
Nucleotide excision repair	3.89E -04
Mismatch repair	4.08E -03
Pyrimidine metabolism	2.14E -03

**Table 3:** Differentially expressed genes.

Name	Biological process related	Gene expression ratio(simulated microgravity/untreated control)	Trend of change
*BBC3*	DNA damage response	2.038	U
*PCNA*	regulation of DNA replication; DNA repair	0.378	D
*P11388-2*	DNA replication; DNA repair	0.4042	D
*TYMS*	DNA replication; DNA repair	0.4021	D
*RFC2*	DNA replication	0.4927	D
*GMNN*	negative regulation of DNA replication	0.4927	D
*MCM7*	DNA replication	0.4761	D
*FEN1*	DNA replication	0.4676	D
*POLE3*	DNA replication	0.4636	D
*GINS2*	DNA replication	0.4492	D

U-Up regulate.

D-Down regulate.

It can be seen from the results above, that the replication of DNA is inhibited under simulated microgravity, but the response to DNA damage is enhanced, and the repair of DNA is reduced at the same time, which leads to the observable chromosome breakage, and the expression of chromosome fragile site. The expression rate of chromosome fragile site increases after the cells were kept under simulated microgravity for 72 hours, and it is therefore believed that the structural chromosome instability of human PBL cells is enhanced under simulated microgravity.

## Conclusions

**Figure 9:**
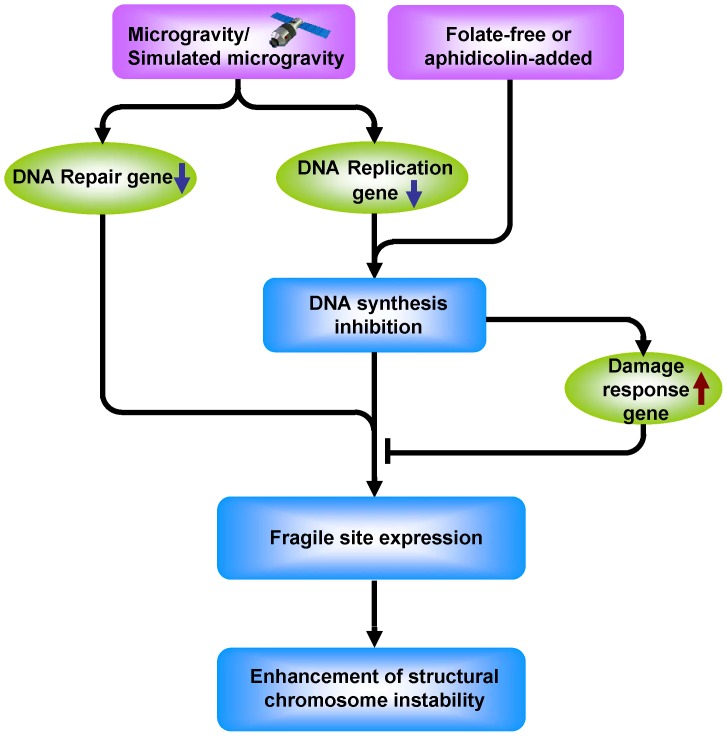
Possible pathway of DNA replication is inhibited which leads to the enhancement of structural chromosome instability under simulated microgravity.

As shown in Fig.9, after the human PBL cells were kept under simulated microgravity for 72 hours in a RCCS bioreactor, 1) the number of chromosomes remains unchanged in different culture media, and no structural change can be detected in the serum added 1640 medium, but the expression of *ATR* in human PBL is enhanced together with the inhibition of cell proliferation; 2) the expression rate of chromosome fragile site is enhanced by inducing the chromosome fragile site with conditioned medium under simulated microgravity for 72 hours; 3) the expression of DNA replication and the DNA repair genes are both down regulated; and 4) the replication of DNA is inhibited, which enhances the structural chromosome instability of human PBL cells under simulated microgravity.

It can therefore be concluded that simulated microgravity has no effect on the numerical chromosome instability of human PBL cells,but it enhances the structural chromosome instability of human PBL cells through the inhibition of DNA replication and the reduction of DNA repair. So, the mechanism of chromosome fragile site induced by simulated microgravity can be explained using the changes which occur in the chromosome structure of human PBL cells, the DNA replication and repair under simulated microgravity.

## Supporting Information

Figure S1 **Morphology of chromosome in PBL cells after the cells were kept under simulated microgravity for 72 hours.** The number of chromosomes was counted using a conventional chromosome analysis technique, and it did not show any significant change. a) Structure of chromosome in PBL cells after the cells are cultured with normal medium (serum added 1640 medium) under 1 *g* condition. b) There is no change in chromosome structure and no chromosome fragile site is observed under simulated microgravity for 72 hours. c-d) Expression of chromosome fragile site in PBL cells after the cells were kept in conditioned medium under simulated microgravity. The blood collected from the volunteers was cultured in folate-free M199 and 0.4 µM aphidicolin added medium for 26 hours before the cells were harvested. Arrows point to chromosome gaps which can be clearly seen both in untreated control (c) and simulated microgravity (d).
(TIF)

Table S1 **Occurrence of Chromosome fragile site observed in 100 cells kept under simulated microgravity for 72 hours.**
(DOC)

Table S2 **Expression rate of chromosome fragile site under simulated microgravity.**
(DOC)

Table S3 **Gene expression ratio obtained through qRT-PCR and microarray.**
(DOC)
