# Supplementary figures and images for: Experimental Study on Effect of Simulated Microgravity on Structural Chromosome Instability of Human Peripheral Blood Lymphocytes
Source: PLoS One. 2014 Jun 25;9(6):e100595. doi: 10.1371/journal.pone.0100595 (PMC4070949; doi:10.1371/journal.pone.0100595)

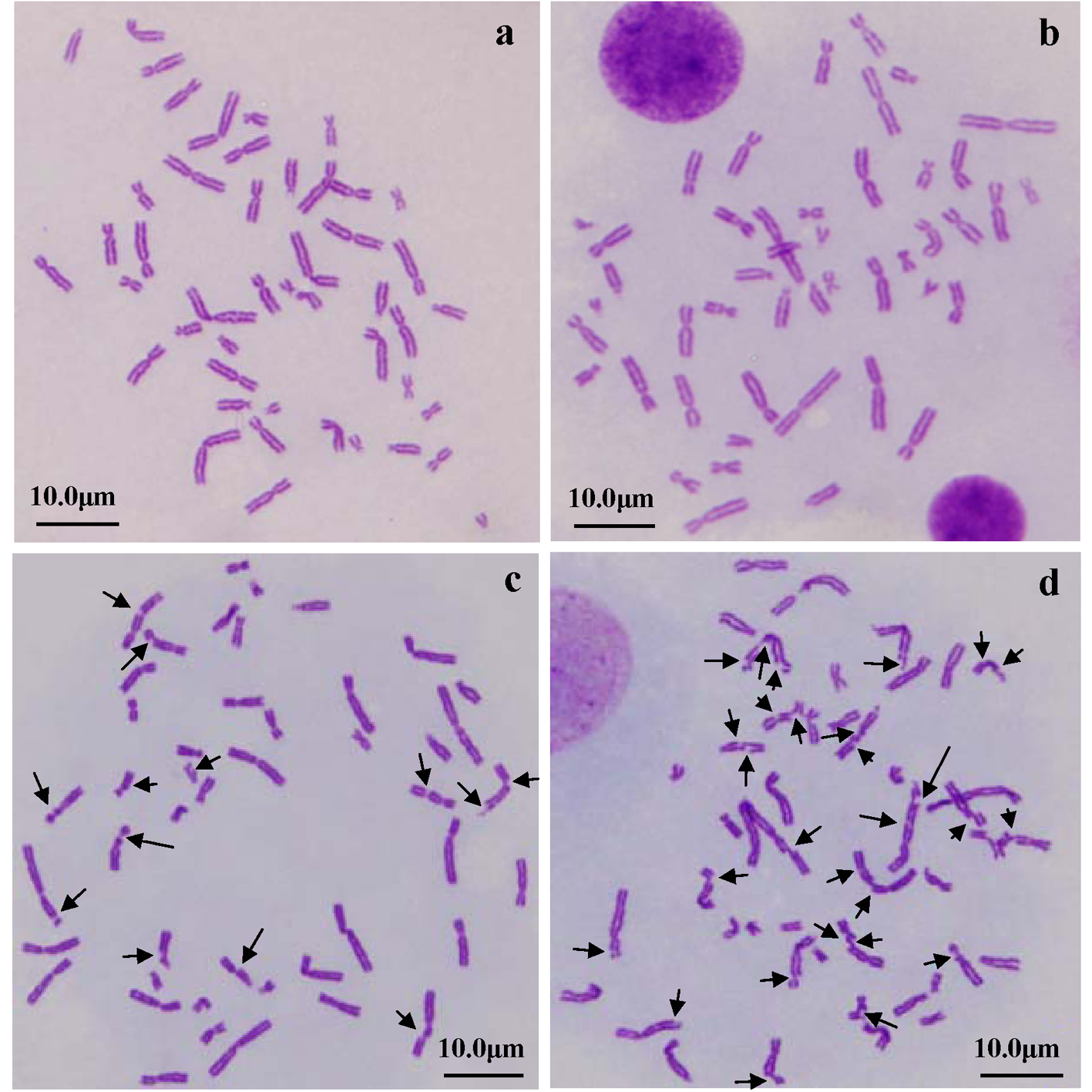

Supplement: Figure S1 — Morphology of chromosome in PBL cells after the cells were kept under simulated microgravity for 72 hours. The number of chromosomes was counted using a conventional chromosome analysis technique, and it did not show any significant change. a) Structure of chromosome in PBL cells after the cells are cultured with normal medium (serum added 1640 medium) under 1 g condition. b) There is no change in chromosome structure and no chromosome fragile site is observed under simulated microgravity for 72 hours. c-d) Expression of chromosome fragile site in PBL cells after the cells were kept in conditioned medium under simulated microgravity. The blood collected from the volunteers was cultured in folate-free M199 and 0.4 µM aphidicolin added medium for 26 hours before the cells were harvested. Arrows point to chromosome gaps which can be clearly seen both in untreated control (c) and simulated microgravity (d). (TIF) [file pone.0100595.s001.tif]
