# Supplementary material for: Experimental Study on Effect of Simulated Microgravity on Structural Chromosome Instability of Human Peripheral Blood Lymphocytes
Source: PLoS One. 2014 Jun 25;9(6):e100595. doi: 10.1371/journal.pone.0100595 (PMC4070949; doi:10.1371/journal.pone.0100595)
Supplement: Table S1 — Occurrence of Chromosome fragile site observed in 100 cells kept under simulated microgravity for 72 hours. (DOC) [file pone.0100595.s002.doc]

Table S1 Occurrence of Chromosome fragile site observed in 100 cells kept under simulated microgravity for 72 hours

| Sample No. | Treatment | Total number of fragile sites observed in 100 cells | Number of fragile sites observed on Chromosome 1 in 100 cells | Number of fragile sites observed on Chromosome 2 in 100 cells |
| --- | --- | --- | --- | --- |
| 1 | CK | 1865 | 43 | 39 |
|  | SMG | 4353* | 67* | 50* |
| 2 | CK | 2084 | 38 | 30 |
|  | SMG | 4279* | 56* | 42* |
| 3 | CK | 1973 | 35 | 35 |
|  | SMG | 4081* | 58* | 48* |
| 4 | CK |  | 45 | 42 |
|  | SMG |  | 72* | 65* |

CK- Untreated control; SMG- Simulated microgravity

*0.01<*P*<0.05, ***P*<0.01, and ****P*<0.001 ($\chi^{2}$-test as compared to untreated control group).
