# Supplementary material for: Experimental Study on Effect of Simulated Microgravity on Structural Chromosome Instability of Human Peripheral Blood Lymphocytes
Source: PLoS One. 2014 Jun 25;9(6):e100595. doi: 10.1371/journal.pone.0100595 (PMC4070949; doi:10.1371/journal.pone.0100595)
Supplement: Table S2 — Expression rate of chromosome fragile site under simulated microgravity. (DOC) [file pone.0100595.s003.doc]

Table S2 Expression rate of chromosome fragile site under simulated microgravity

| Sample No. | Treat-ment | FRA3B | | FRA11F | | FRA16D | |
| --- | --- | --- | --- | --- | --- | --- | --- |
|  |  | Total number of fragile sites observed in 100 cells | Expression rate of chromosome fragile site (%) | Total number of fragile sites observed in 100 cells | Expression rate of Chromosome fragile site (%) | Total number of fragile sites observed in 100 cells | Expression rate of chromosome fragile site (%) |
| 1 | CK | 77 | 38.5 | 30 | 15 | 51 | 25.5 |
|  | SMG | 113 | 56.5** | 43 | 21.5** | 71 | 35.5** |
| 2 | CK | 68 | 34 | 26 | 13 | 45 | 22.5 |
|  | SMG | 105 | 52.5** | 37 | 18.5** | 70 | 35** |
| 3 | CK | 79 | 39.5 | 20 | 10 | 55 | 27.5 |
|  | SMG | 121 | 60.5** | 31 | 15.5** | 81 | 40.5** |
| 4 | CK | 74 | 37 | 28 | 14 | 40 | 20 |
|  | SMG | 109 | 54.5** | 39 | 19.5** | 68 | 34** |

*0.01<*P*<0.05, ***P*<0.01, and ****P*<0.001 ($\chi^{2}$-test as compared to untreated control group).
