# Supplementary material for: Experimental Study on Effect of Simulated Microgravity on Structural Chromosome Instability of Human Peripheral Blood Lymphocytes
Source: PLoS One. 2014 Jun 25;9(6):e100595. doi: 10.1371/journal.pone.0100595 (PMC4070949; doi:10.1371/journal.pone.0100595)
Supplement: Table S3 — Gene expression ratio obtained through qRT-PCR and microarray. (DOC) [file pone.0100595.s004.doc]

Table S3 Gene expression ratio obtained through qRT-PCR and microarray

| Gene | qRT-PCR | Microarray |
| --- | --- | --- |
| *IFI27* | 3.85 | 2.0538 |
| *CDC2* | 0.4158 | 0.4368 |
| *KIF2C* | 0.56479 | 0.4338 |
| *MAD2L1* | 0.6212 | 0.4025 |
| *PCNA* | 0.6360 | 0.378 |
| *CCNA2* | 0.609 | 0.3516 |

Note: The gene expression ratio in 10 samples obtained through qRT-PCR and microarray shows the same tendency of up regulate and down regulate, which means the results of microarray are credible.
